# *Plasmodium falciparum*, anaemia and cognitive and educational performance among school children in an area of moderate malaria transmission: baseline results of a cluster randomized trial on the coast of Kenya

**DOI:** 10.1111/j.1365-3156.2012.02971.x

**Published:** 2012-04-19

**Authors:** Katherine E Halliday, Peris Karanja, Elizabeth L Turner, George Okello, Kiambo Njagi, Margaret M Dubeck, Elizabeth Allen, Matthew CH Jukes, Simon J Brooker

**Affiliations:** 1 Faculty of Infectious and Tropical Diseases, London School of Hygiene and Tropical MedicineUK; 2 Malaria Public Health and Epidemiology Group, Kenya Medical Research Institute-Wellcome Trust Collaborative ProgrammeNairobi, Kenya; 3 Faculty of Epidemiology and Public Health, London School of Hygiene and Tropical MedicineUK; 4 Division of Malaria Control, Ministry of Public Health and SanitationNairobi, Kenya; 5 Department of Teacher Education, College of CharlestonSC, USA; 6 Graduate School of Education, Harvard UniversityCambridge, MA, USA

**Keywords:** malaria, *plasmodium*, anaemia, sustained attention, cognition, educational achievement, school children, Kenya

## Abstract

**Objectives:**

Studies have typically investigated health and educational consequences of malaria among school-aged children in areas of high malaria transmission, but few have investigated these issues in moderate transmission settings. This study investigates the patterns of and risks for *Plasmodium falciparum* and anaemia and their association with cognitive and education outcomes on the Kenyan coast, an area of moderate malaria transmission.

**Methods:**

As part of a cluster randomised trial, a baseline cross-sectional survey assessed the prevalence of and risk factors for *P. falciparum* infection and anaemia and the associations between health status and measures of cognition and educational achievement. Results are presented for 2400 randomly selected children who were enrolled in the 51 intervention schools.

**Results:**

The overall prevalence of *P. falciparum* infection and anaemia was 13.0% and 45.5%, respectively. There was marked heterogeneity in the prevalence of *P. falciparum* infection by school. In multivariable analysis, being male, younger age, not sleeping under a mosquito net and household crowding were adjusted risk factors for *P. falciparum* infection, whilst *P. falciparum* infection, being male and indicators of poor nutritional intake were risk factors for anaemia. No association was observed between either *P. falciparum* or anaemia and performance on tests of sustained attention, cognition, literacy or numeracy.

**Conclusion:**

The results indicate that in this moderate malaria transmission setting, *P. falciparum* is strongly associated with anaemia, but there is no clear association between health status and education. Intervention studies are underway to investigate whether removing the burden of chronic asymptomatic *P. falciparum* and related anaemia can improve education outcomes.

## Introduction

The health of school children has received increasing attention over the last two decades, and there are increased efforts to implement school health programmes, delivering anthelmintics and micronutrients (Bundy *et al.* 2006; Bundy 2011). Less emphasis has been given to malaria as a health problem facing school children despite them experiencing some of the highest age-specific rates of *Plasmodium falciparum* infection (Smith *et al.* 2007; Hay *et al.* 2008; Brooker *et al.* 2009), which can have a number of direct and indirect consequences, including anaemia (Draper 1960). The control of malaria is associated with significant improvements in haemoglobin levels among both young children (Korenromp *et al.* 2004) and children of school age (Draper 1960; Geerligs *et al.* 2003; Clarke *et al.* 2008; Barger *et al.* 2009). Malaria may have additional consequences for children’s cognitive performance and ultimately educational achievement (Al Serouri *et al.* 2000; Holding & Snow 2001; Kihara *et al.* 2006; Vitor-Silva *et al.* 2009; Fernando *et al.* 2010). For instance, malaria has been related to increased absenteeism (Brooker *et al.* 2000; Fernando *et al.* 2003a; Kimbi *et al.* 2005), grade repetition (Thuilliez 2010) and poorer educational achievement (Fernando *et al.* 2003a, b). Studies in Ghana, Kenya and Sri Lanka suggest that malaria prevention can improve school attendance, sustained attention and educational achievement (Colbourne 1955; Fernando *et al.* 2006; Jukes *et al.* 2006; Clarke *et al.* 2008).

The consequences of malaria for school children and the benefits of school-based malaria control are likely to vary in different settings, particularly according to intensity of malaria transmission and the relative contribution of other causes of anaemia and poor education outcomes. A previous study in an area of perennial high malaria transmission in western Kenya (Clarke *et al.* 2008) investigated the impact of intermittent preventive treatment for malaria in schools and found a large impact on children’s concentration in class and a 48% reduction in the rates of anaemia. However, no effect on educational achievement was observed. To investigate this result further and find out whether the benefits of malaria control are observed in settings with different intensities of malaria transmission and different educational standards, an ongoing cluster randomised trial is investigating the impact of an alternative school-based malaria intervention, intermittent screening and treatment (IST), in an area of moderate malaria transmission on the coast of Kenya (Brooker *et al.* 2010). The current paper presents data from the baseline cross-sectional survey of this trial and investigates risk factors for *P. falciparum* infection and anaemia as well as correlates of cognition, attention and educational achievement.

## Methods

The study design and methods of the intervention trial have been previously detailed (Brooker *et al.* 2010) and are briefly summarized below. The IST intervention under investigation in the trial comprises mobile health teams consisting of laboratory technicians and nurses visiting schools each term. Children are asked to provide a finger prick blood sample to test for the presence of malaria parasites using ParaCheck-*Pf* malaria rapid diagnostic tests (RDT) (Orchid Biomedical Systems, Goa, India). Children (with or without malaria symptoms) found to be RDT-positive are treated with artemether-lumefantrine (Coartem®, Novartis), an artemisinin-based combination therapy. The current investigation uses baseline cross-sectional data collected between February and March 2010 in the 51 intervention schools that were allocated to receive the intermittent screening and treatment for malaria. No baseline data were collected on *P. falciparum* infection for the 50 control schools not receiving the malaria intervention owing to ethical considerations about screening for malaria and not providing treatment. Results reported here on *P. falciparum* infection are based on expert microscopy. The reliability of the RDTs employed for the IST will be evaluated in future analyses.

Reporting of the current study has been verified in accordance with the STROBE (Strengthening the Reporting of Observational Studies in Epidemiology) checklist.

### Study setting

The surveys were conducted in Kwale and Msambweni districts on the south Kenyan coast, where malaria transmission is seasonal following the two rainy seasons (April-July and September-November) (Snow *et al.* 1993). In economic and educational terms, the districts are ranked the seventh poorest in Kenya and consistently have some of the worst-performing schools in the national school examinations (RTI International, 2008). In 2009, mass albendazole treatment was provided to all schools as part of the national school deworming programme. No systematic treatment for schistosomiasis has been provided to date and no specific school-based malaria control efforts are ongoing.

### Recruitment

A school census of all 101 schools included in the trial was conducted by trained personnel and was used as a sampling frame from which 25 children from class 1 and 30 children from class 5 were randomly selected using random number tables. Fewer children were selected from class 1 because of the extra educational assessments undertaken with these children and the feasibility of conducting the tests in a single day.

### Health surveys

Finger prick blood samples were obtained from all children to assess haemoglobin concentration (Hb) using a portable haemoglobinometer (Hemocue, Ängelholm, Sweden). Children with severe anaemia (Hb < 80 g/l) were referred to the nearest health facility for iron therapy as per the national guidelines. Height and weight were measured and axillary temperature was digitally recorded. Thick and thin blood smears, prepared for malaria microscopy, were stained with 2% Giemsa for 30 minutes Parasite densities were determined from thick blood smears by counting the number of asexual parasites per 200 white blood cells, assuming a white blood cell count of 8000/μl. A smear was considered negative after reviewing 100 high-powered fields. Thin blood smears were reviewed for species identification. Two independent microscopists read the slides, with a third microscopist resolving discrepancies.

### Assessments of cognition and educational achievement

These have been detailed elsewhere (Brooker *et al.* 2010). In brief, age-appropriate tests of sustained attention were conducted in each class: the pencil-tap test and the code transmission test adapted from the Test of everyday attention for children (TEA-Ch) group (Manly *et al.* 1999) for class 1 and class 5 children, respectively. Non-verbal reasoning was assessed in class 1 by the Raven’s Progressive Matrices task (Raven *et al.* 1998). A range of class-specific literacy and numeracy tests were conducted in individualized and small-group settings. The tests were extensively piloted and adapted to the context. Test–retest reliabilities of 0.7 were required for inclusion of tests in the battery.

### Risk factors

During the informed consent process, a questionnaire was administered to parents/guardians to record household information on residence, family size, ownership of possessions, mosquito net use by them and their children, recent deworming of the child, house construction and education level of the parent. For children in class 1, additional information on household literacy, the language spoken in the household and reading practices was recorded. At each school, a questionnaire was administered to the head teacher to collect information on school demography, sanitation facilities, presence of school feeding and other health programmes. School locations were mapped using a Global Positioning System (GPS) receiver (eTrex Garmin Ltd., Olathe, KS, USA). Elevation of schools was recorded and used as a geographical marker of distance from the coast.

### Statistical analysis

Data were double-entered using customized data entry screens in Microsoft Access (Microsoft Corporation, Seattle, USA). Consistency checks were performed and all discrepancies and queries were verified against the original paper forms. Health data were linked by school location and mapped using ArcGIS 9.3.1 (Environmental Systems Research Institute Inc., Redlands, CA, USA).

*Plasmodium falciparum* infection was defined on the basis of duplicate slide readings. Anaemia was defined using age- and sex-corrected WHO thresholds (Benoist *et al.* 2008), with no correction made for altitude. The anthropometric indices, z-scores of height-for-age (HAZ), weight-for-age (WAZ) and body mass index-for-age (BMIZ), were calculated using the AnthroPlus software for children aged 5–19 years (WHO, 2007), assuming a mid-year age for each child because of doubts over the correct date of birth. Weight-for-age z-score was only calculated for children aged 5–10 years. Children were classified as stunted, underweight or thin if HAZ, WAZ and BMIZ, respectively, were less than two standard deviations below the reference median. Age of the children was provided by themselves and by their parents. Ages provided by the children were used to calculate anaemia and anthropometric indices as they were considered more reliable. A sensitivity analysis using parent-reported ages for all multivariable models indicated minimal sensitivity. Age was modelled as a categorical variable for the *P. falciparum* and anaemia risk factor analyses and as a continuous variable for the attention and education analyses owing to the smaller age ranges observed once stratified by class. Household asset data were used to derive an index of socio-economic status (SES), based on the entire trial population. The principal component analysis (PCA) approach proposed by Filmer and Pritchett (Filmer & Pritchett 2001) was used. Variables included into the PCA included ownership of a bicycle, motorcycle, mobile phone, radio, television, as well as presence of electricity, pit latrine, and brick and cement construction materials. The first principal component explained 30.6% of the overall variability and gave greatest weight to the household construction materials followed by ownership of a television. The resultant scores were divided into quintiles so that households could be classified according to relative SES. No internal validation of the index was undertaken. Finally, elevation (a proxy for distance from the coastline) was divided into tertiles.

Analyses were performed using STATA version 11.0 (STATA Corporation, College Station, TX, USA). The outcomes of interest examined were prevalence of *P. falciparum* and of anaemia (binary outcomes) and scores for spelling, number identification, numeracy, comprehension, code transmission and pencil-tapping tasks (continuous outcomes). Univariable associations between the health-related outcomes and risk factors were assessed using multilevel logistic regression, accounting for school-level clustering (Rabe-Hesketh & Skrondal 2008). Variables demonstrating an association at the 10% significance level were subsequently included into a multivariable, multilevel logistic regression model, accounting for school-level clustering. Stepwise elimination was used to create the final model using a 5% significance level for retention in the model. Age and sex were treated as *a priori* risk factors and retained in multivariable models. *A priori* interactions between net use with age and sex and between school feeding and elevation (distance from the coast) were investigated.

Analysis of the cognitive and education outcomes was stratified by class and focused on associations with *P. falciparum* infection and with anaemia, additionally accounting for age and sex as *a priori* risk factors. For the pencil-tap assessment of sustained attention in class 1 children, the analysis was split into two because of the significant proportion of children who were disengaged and scored zero. The proportion of children engaged in the task was examined by different variables using multilevel logistic regression accounting for school-level clustering. For each of the spelling assessments in classes 1 (score 0–20) and 5 (score 0–43); the numeracy in classes 1 (score 0–20) and 5 (score 0–38); the Ravens assessment in class 1 (score 0–20); the sentence comprehension in class 5 (score 0–40); the code transmission assessment of sustained attention in class 5 children (score 0–20); and the analysis of children who were engaged in the pencil-tap task (score 1–20), the effect of explanatory variables was quantified by mean differences in test performance using linear regression. Bootstrapping was used to account for non-normality of the scores, whereby schools were resampled to account for school-level clustering (Efron & Tibshirani 1994). Bias-corrected confidence intervals based on the bootstrap resamples were obtained. Significant (*P* < 0.1) variables identified in univariable analysis were considered for the multivariable model which employed stepwise elimination.

### Ethical considerations

The study was approved by the Kenya Medical Research Institute and National Ethics Review Committee (SSC No. 1543), the London School of Hygiene and Tropical Medicine Ethics Committee (5503), and the Harvard University Committee on the Use of Human Subjects in Research (F17578-101). Meetings were held in participating schools to explain the nature and purpose of the trial to parents or legal guardians, and written informed consent was obtained.

## Results

**Figure 1:**
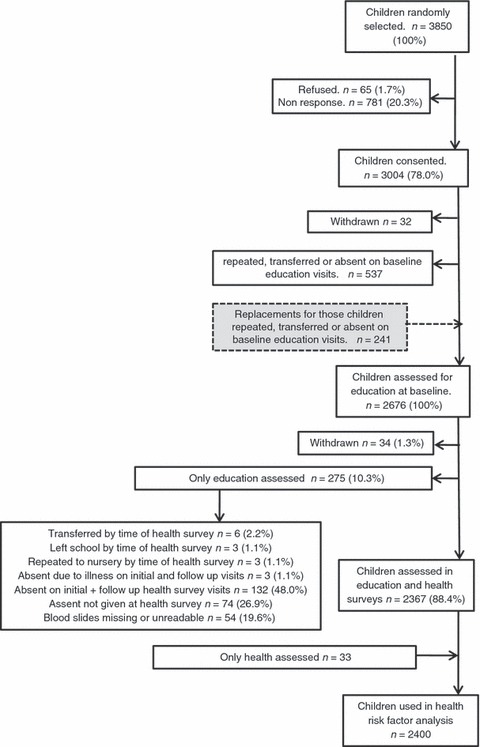
Data flow diagram for the education and health surveys conducted in school children in 51 schools on the south coast of Kenya, 2010.

Of the 3850 children randomly selected to participate in the study, 2400 (1160 in class 1 and 1240 in class 5) were included in the analysis (Figure 1), with a mean of 48 children per school (range: 26–60). No systematic differences in individual and household characteristics were observed between included children and those children excluded because of missing health data (Table S1, online-only). The mean age of children in the present analysis was 10.3 years (range: 5–18 years) and the male/female ratio was 0.95 (Table 1).

**Table 1:** Univariable analysis for associations between *Plasmodium falciparum* infection and anaemia and potential risk factors for both health outcomes among school children in 51 schools on the south coast of Kenya, 2010.

Variable	Number of children (%)* *n* = 2400	Number of children (%) with *Plasmodium falciparum n* = 311	Odds ratio (95% CI)	*P*-value†	Number of children (%) with anaemia *n* = 1091	Odds ratio (95% CI)	*P*-value†
**Child-level**							
Sex							
Male	1167 (48.6)	173 (14.8)	1		566 (48.5)	1	0.001
Female	1233 (51.4)	138 (11.2)	0.67 (0.51–0.87)	0.003	525 (42.6)	0.76 (0.65–0.90)	
Age (per additional year)‡							
	10.34 (2.81)		0.92 (0.87–0.96)	<0.001		1.00 (0.97–1.03)	0.863
Age groups (years)							
5–9	940 (39.2)	139 (14.8)	1		458 (48.7)	1	0.002
10–12	830 (34.6)	120 (14.5)	0.90 (0.67–1.21)		337 (40.6)	0.71 (0.59–0.87)	
13–18	630 (26.2)	52 (8.3)	0.42 (0.29–0.61)	<0.0001	296 (47.0)	0.96 (0.78–1.18)	
*Plasmodium falciparum* infection status							
Not infected	2089 (87.0)				914 (43.8)	1	<0.001
Infected	311 (13.0)	–	–		177 (56.9)	1.66 (1.28–2.15)	
*Plasmodium falciparum* density (p/μl)							
No infection (0)	2089 (87.0)				914 (43.8)	1	<0.0001
Low (1–999)	237 (9.9)	–	–		124 (52.3)	1.37 (1.03–1.82)	
Medium/high (≥1000)	74 (3.1)				53 (71.6)	3.28 (1.93–5.57)	
WAZ (z-scores)§,¶,**							
Not wasted	709 (75.8)	102 (14.4)	1		343 (48.4)	1	0.726
Wasted	227 (24.2)	37 (16.3)	1.37 (0.86–2.17)	0.188	113 (49.8)	1.06 (0.77–1.46)	
HAZ (z-scores)§							
Not stunted	1790 (74.8)	223 (12.5)	1		794 (44.4)	1	0.016
Stunted	603 (25.2)	88 (14.6)	1.31 (0.97–1.76)	0.083	294 (48.8)	1.27 (1.04–1.54)	
BMIZ (z-scores)§							
Not thin	1950 (81.5)	262 (13.4)	1		895 (45.9)	1	0.363
Thin	442 (18.5)	49 (11.1)	0.85 (0.60–1.22)	0.377	193 (43.7)	0.91 (0.73–1.12)	
Child been dewormed in last year¶							
No	442 (19.5)	72 (16.3)	1		181 (41.0)	1	0.13
Yes	1824 (80.5)	220 (12.1)	0.70 (0.50–0.97)	0.033	843 (46.2)	1.19 (0.95–1.48)	
**Household-level**							
Education level of household head							
No schooling	814 (34.3)	131 (16.1)	1		392 (48.2)	1	0.047
Primary	1228 (51.8)	153 (12.5)	0.83 (0.63–1.11)		523 (42.6)	0.79 (0.66–0.95)	
Secondary	255 (10.8)	17 (6.7)	0.50 (0.28–0.89)	0.056	126 (49.4)	1.05 (0.78–1.41)	
College/degree	74 (3.1)	6 (8.1)	0.54 (0.22–1.34)		34 (46.0)	0.91 (0.56–1.50)	
Water source							
Uncovered (stream/river/dam)	318 (13.4)	63 (19.8)	1		135 (42.5)	1	0.742
Covered (well/borehole/piped)	2066 (86.7)	246 (11.9)	0.76 (0.49–1.20)	0.24	948 (45.9)	1.05 (0.80–1.38)	
SES							
Poorest	577 (24.2)	89 (15.4)	1		270 (46.8)	1	0.207
Poor	504 (21.1)	67 (13.3)	0.94 (0.64–1.38)		240 (47.6)	1.01 (0.79–1.29)	
Median	423 (17.7)	61 (14.4)	1.00 (0.67–1.48)	0.206	171 (40.4)	0.75 (0.58–0.98)	
Less poor	459 (19.3)	48 (10.5)	0.83 (0.55–1.27)		206 (44.9)	0.89 (0.69–1.15)	
Least poor	422 (17.7)	44 (10.4)	0.66 (0.42–1.03)		197 (46.7)	0.93 (0.72–1.22)	
Number of people in the house‡							
	7.06 (2.52)	–	1.06 (1.00–1.12)	0.036	–	1.01 (0.98–1.05)	0.39
Number of children in the house‡							
	4.82 (2.18)	–	1.04 (0.98–1.11)	0.183	–	1.03 (0.99–1.07)	0.196
Child sleeps under a net							
No	880 (37.2)	140 (15.9)	1		406 (46.1)	1	0.554
Yes	1489 (62.8)	166 (11.2)	0.62 (0.47–0.82)	<0.001	666 (44.7)	0.95 (0.80–1.13)	
If yes, is the net treated?							
No	278 (8.7)	34 (12.2)	1		129 (46.4)	1	0.643
Yes	1161 (78.2)	127 (11.0)	1.06 (0.67–1.68)	0.879	518 (44.6)	0.93 (0.71–1.22)	
Don’t know	46 (3.1)	5 (10.9)	0.83 (0.27–2.58)		18 (39.1)	0.74 (0.39–1.41)	
Number of nets in the house¶							
No nets	360 (17.0)	55 (15.3)	1		170 (47.2)	1	0.423
1–2 nets	655 (30.9)	98 (15.0)	1.01 (0.67–1.50)		286 (43.7)	0.84 (0.64–1.10)	
3–4 nets	810 (38.2)	85 (10.5)	0.65 (0.43–0.99)	0.003	362 (44.7)	0.90 (0.69–1.17)	
≥5 nets	295 (13.9)	26 (8.8)	0.46 (0.27–0.80)		136 (46.1)	0.96 (0.70–1.33)	
**School-level**							
School malaria control activities							
No	1814 (75.6)	238 (13.1)	1		835 (46.0)	1	0.531
Yes	586 (24.4)	73 (12.5)	1.20 (0.48–3.00)	0.697	256 (43.7)	0.90 (0.66–1.24)	
School feeding programme							
No	1115 (46.5)	139 (12.5)	1		555 (49.8)	1	0.017
Yes	1285 (53.5)	172 (13.4)	0.89 (0.40–1.97)	0.775	536 (41.7)	0.73 (0.57–0.94)	
Elevation (metres)							
0–50	708 (29.5)	120 (17.0)	1		370 (52.3)	1	0.013
51–100	919 (38.3)	119 (13.0)	0.59 (0.24–1.50)		410 (44.6)	0.73 (0.54–0.99)	
101–200	773 (32.2)	72 (9.3)	0.39 (0.15–1.05)	0.176	311 (40.2)	0.61 (0.45–0.84)	

* Displayed as number and percentage except for continuous variables, displayed as mean and standard deviation (SD).

† *P*-value is from likelihood ratio test comparing multilevel logistic regression models (adjusting for school-level clustering), with and without character of interest.

‡ Modelled as continuous variable.

§ WAZ – weight-for-age HAZ – height-for-age BMI – body mass index-for-age. Wasted, stunted and thin defined as WAZ, HAZ and BMIZ z-scores < 2 SD.

¶ Characteristics with missing data vary (all below 2% missing except deworming 5.6% missing, number of nets owned 11.7% missing, and WAZ 61% missing).

** WAZ only calculated for children aged 5 years to 10 years.

### *Plasmodium falciparum* and anaemia

**Figure 2:**
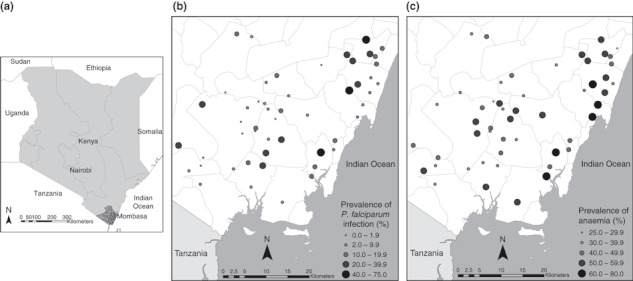
(a) The location of the study site in Kenya. (b) The geographical distribution of *Plasmodium falciparum* infection in 51 schools on the south coast of Kenya, 2010. (c) The geographical distribution of anaemia (adjusted for age and sex) in 51 schools on the south coast of Kenya, 2010.

The overall prevalence of *P. falciparum* was 13.0% (95% confidence interval [CI]: 8.9–17.0%); only 11 infected children had documented fever. Infection prevalence varied markedly by school, ranging from 0 to 75.0% (Figure 2b), with no infected children found in seven schools and a prevalence exceeding 40% in three schools. Overall, 45.5% (95% CI: 42.0–48.9%) of children were anaemic, and 1.1% (95% CI: 0.7–1.5) were severely anaemic. The mean haemoglobin concentration was 117.5g/l (95% CI: 116.4–118.6). Marked heterogeneity was also observed in the school-level prevalence of anaemia (range: 26.3–80.0%) (Figure 2c).

### Risk factors for *Plasmodium falciparum* infection and anaemia

The relative frequencies of individual, household and school-level risk factors for *P. falciparum* infection and anaemia are shown in Table 1. Overall, 62.8% (95% CI: 58.7–67.0) of children reported sleeping under a mosquito net, but usage varied markedly by school (range: 26.4–93.3%). In univariable analysis, *P. falciparum* infection was significantly associated with being male, younger age, stunting, absence of deworming, education level of household head, increased number of people in the household, fewer mosquito nets in the household and not sleeping under a mosquito net. In the multivariable model, higher odds of *P. falciparum* infection were significantly associated with being male, younger age groups, increasing number of people living in the child’s household and the child not sleeping under a net (Table 2).

**Table 2:** Multivariable risk factor analysis for *Plasmodium falciparum* infection and anaemia among school children in 51 schools on the south coast of Kenya, 2010

Variable	*P. falciparum* infection*			Anaemia†		
	
	Adjusted odds ratio‡	95% confidence interval	*P*-value§	Adjusted odds ratio‡	95% confidence interval	*P*-value§
Sex						
Male	1			1		
Female	0.68	0.51–0.89	0.005	0.8	0.67–0.95	0.009
Age (years)						
5–9	1			1		
10–12	0.87	0.64–1.18		0.71	0.58–0.87	
13–18	0.37	0.25–0.54	<0.001	0.97	0.78–1.20	0.002
Child sleeps under a net						
No	1					
Yes	0.6	0.45–0.79	<0.001	–	–	–
Number of people in the house¶						
	1.07	1.01–1.14	0.014	–	–	–
*P. falciparum* density (p/μl)						
No infection				1		
Low (1–999)				1.41	1.05–1.89	
Medium/high (≥1000)	–	–	–	3.68	2.12–6.38	<0.001
HAZ (z-scores)						
Not stunted				1		
Stunted	–	–	–	1.26	1.03–1.54	0.022
Education level of household head						
No schooling				1		
Primary	–	–	–	0.78	0.64–0.94	
Secondary				1.12	0.83–1.50	0.014
College/degree				0.89	0.53–1.48	
Effect of elevation (metres) by absence/presence of school feeding programme**,§§						
No school feeding						
0–50				1		
51–100				0.58	0.40–0.83	
101–200				0.58	0.34–1.00	
School feeding						
0–50				1		0.003‡‡
51–100				1.3	0.79–2.15	
101–200				1.32	0.63–2.76	
Effect of school feeding programme by elevation (metres)**,††						
0–50						
No school feeding				1		
School feeding				0.46	0.28–0.76	
51–100						
No school feeding				1		
School feeding				1.05	0.72–1.51	0.003‡‡
101–200						
No school feeding				1		
School feeding				0.82	0.48–1.39	

* *n* = 2369 observations included for children with complete data for all variables.

† *n* = 2364 observations included for children with complete data for all variables.

‡ Adjusted for variables included in final multivariable regression model as shown.

§ *P*-value derived from likelihood ratio test in multivariable multilevel, logistic regression model, adjusted for school-level clustering.

¶ Modelled as continuous variable.

** There was statistical evidence of an interaction between elevation of schools and presence of a school feeding programme on anaemia; therefore, the stratum-specific results are reported for both school feeding and elevation (*P*-value derived from likelihood ratio test comparing the model with school feeding and elevation variables separately with the model also including the interaction between the two variables is *P* = 0.042).

‡‡ *P*-value is derived from likelihood ratio test comparing the model with both the school feeding and elevation variables and their interaction term with the model without either of the variables.

§§ Of those children without access to a school feeding programme, 515 attend schools at elevations of 0–50m, 442 attend schools at elevations of 51–100m, 137 attend schools at elevations of 101–200m. Of those children with access to a school feeding programme, 176 attend schools at elevations of 0–50m, 466 attend schools at elevations of 51–100m, 628 attend schools at elevations of 101–200m.

†† At elevation group 50–100 m, 176 children have school feeding and 515 do not. At group 51–100 m, 466 children have school feeding and 442 do not. At elevation group 101–200 m, 628 children have school feeding and 137 do not.

In univariable analysis, anaemia was significantly associated with male sex, younger age, *P. falciparum* infection, being stunted, education level of household head, not attending a school with an active school feeding programme and attending school at lower elevation, closer to the coast. In multivariable analysis, increased odds of anaemia were significantly associated with *P. falciparum* infection, with the odds increasing with increasing parasite density (AOR [adjusted odds ratio], 3.68; 95% CI: 2.12–6.38 *P* < 0.001) for children with high intensity infection *vs.* those with no infection, and for children who were stunted. Significantly lower odds of anaemia were associated with children who were female, and with being aged 10–12 years old *vs.* 5–9 years old. The effect of a school feeding programme on anaemia was modified by elevation of school (distance from coast) and thus is presented by stratum-specific odds ratios. School feeding was associated with lower odds of anaemia in schools closest to the coast (AOR, 0.46; 95% CI: 0.28–0.76; *P* = 0.003), with no evidence of an association for schools positioned further from the coast.

### Associations with cognition and educational achievement

Results from the univariable analysis of the associations between cognition and educational achievement and health and other factors are presented in online-only Tables S2, S3 and S4. Results from multivariable analyses are presented in Tables 3 (attention and literacy assessments) and 4 (cognitive, comprehension and numeracy assessments), which report significant associations between scores and several child-level variables. In all tasks, increasing age was associated with higher scores among children in class 1, but with lower scores among children in class 5. For several tasks, girls were found to have lower scores than boys. Neither *P. falciparum* infection, irrespective of parasite density, nor anaemia was found to be associated with any cognitive or educational outcome. Interestingly, poor engagement in the attention task for class 1 was associated with eating breakfast and attending a school with school feeding, and better spelling performance in class 5 was found among children who were classified as thin on the basis of BMI.

**Table 3:** Multivariable risk factor analysis – associations of *Plasmodium falciparum* infection and anaemia with a test of sustained attention and a test of literacy in children in classes 1 and 5 on the south coast of Kenya, 2010.

Variable	Attention assessments						Literacy assessments			
	
	Pencil tap – Class 1				Code transmission – Class 5		Spelling – Class 1		Spelling – Class 5	
			
	Adjusted OR for engagement (95% CI) *n* = 1122	*P*-value*	Mean adjusted difference in performance in children who were engaged†,‡(95% CI) *n* = 998	*P*-value§	Mean adjusted difference in performance (95% CI)† *n* = 1227	*P*-value §	Mean adjusted difference in performance (95% CI)† *n* = 1127	*P*-value§	Mean adjusted difference in performance (95% CI)† *n* = 1216	*P*-value§
**Child-level**										
* P. falciparum* density (p/μl)										
No infection (0)	1									
Low (1–999)	1.00 (0.53–1.80)	0.053	−0.05 (−1.01, 1.12)		0.06 (−1.42, 1.52)		0.63 (−0.56, 2.12)		−1.00 (−2.90, 0.89)	
High (≥1000)	6.38 (0.85–47.87)		−0.00 (−1.28, 1.23)	1	−0.97 (−3.40, 1.03)	0.638	1.14 (0.08, 2.28)	0.102	−2.49 (−6.80, 1.31)	0.205
Anaemia status										
Not anaemic	1									
Anaemic	1.20 (0.82–1.77)	0.353	0.17 (−0.41, 0.77)	0.169	0.34 (−0.25, 0.95)	0.25	0.35 (−0.18, 0.98)	0.343	0.69 (−0.28, 1.68)	0.17
Sex										
Male	1									
Female	0.80 (0.55–1.17)	0.249	**−0.62 (−1.16, 0.00)**	**0.037**	−0.61 (−1.37, 0.07)	0.102	0.43 (−0.13, 1.02)	0.268	**−1.32 (−2.14, −0.46)**	**0.003**
Age (years)¶										
	**1.16 (1.03–1.31)**	**0.014**	**0.44 (0.22, 0.62)**	**<0.001**	**−0.30 (−0.47, −0.10)**	**0.003**	**0.27 (0.06, 0.47)**	**0.025**	**−1.32 (−1.65, −1.03)**	**<0.001**
BMIZ (z-score)										
Not thin										
Thin	–	–	–	–	–	–	–	–	**1.12 (0.13, 2.11)**	**0.026**
Eat breakfast before school										
No	**1**		–	–	–	–	–	–	–	–
Yes	**0.46 (0.28–0.74)**	**0.001**								
**Household-level**										
SES quintile										
Poorest										
Poor			**−0.17 (−1.17, 0.72)**				**0.09 (−0.59, 0.91)**		**−0.14 (−1.32, 1.34)**	
Median	–	–	**−0.85 (−1.80, 0.04**)		–	–	**0.49 (−0.33, 1.38)**		**2.30 (1.24, 3.52)**	**<0.001**
Less poor			**−0.92 (−2.00, 0.17)**	0.026			**1.20 (0.43, 2.17)**	**0.006**	**1.42 (0.30, 2.60)**	
Least poor			**−1.27 (−2.33, −0.16)**				**1.48 (0.59, 2.46)**		**3.32 (1.95, 4.80)**	
**School-level**										
School feeding programme										
No	**1**									
Yes	**0.62 (0.39–0.98)**	**0.039**	–	–	–	–	–	–	–	–
Seating in classroom										
Desks or tables and chairs										
Floor	–	–	–	–	–	–	**−1.76 (−3.55, −0.48)**	**0.026**	–	–
Division										
Diani										
Lunga Lunga			**−0.18 (−1.16, 0.74)**						**−3.53 (−5.79, −1.49)**	
Msambweni	–	–	**−1.20 (−2.40, −0.08)**	**0.016**	–	–	–	–	**−3.95 (−6.17, −2.13)**	**<0.001**
Kubo			**−1.58 (−2.78, −0.39)**						**−3.79 (−6.19, −1.33)**	

* *P*-value derived from likelihood ratio test of model with and without variable of interest in multivariable multilevel logistic regression analysis (adjusting for school-level clustering).

† Positive values indicate an increased score over reference group, and negative values indicate a decreased score over reference group (95% CI is the bias-corrected confidence interval).

‡ Only children found to be engaged in task are included.

§ *P*-value is from multivariable Wald test derived from multivariable linear regression, bootstrapped and adjusted for school-level clustering.

¶ Modelled as a continuous variable.

The bold numbers refer to those with a *P*-value of 0.05 or less in the multivariable model.

**Table 4:** Multivariable risk factor analysis – associations of *Plasmodium falciparum* infection and anaemia with a test of cognition and numeracy in children in classes 1 and 5 on the south coast of Kenya, 2010.

Variable	Cognitive non-verbal reasoning assessment		Comprehension assessment		Numeracy assessments			
		
	Ravens test – Class 1		Silly sentences – Class 5		Number identification – Class 1		Written numeracy – Class 5	
			
	Mean difference in performance in children who were engaged (95% CI)* *n* = 1118	*P*-value†	Mean difference in performance (95% CI)* *n* = 1211	*P*-value†	Mean difference in performance (95% CI)* *n* = 1119	*P*-value†	Mean difference in performance(95% CI)* *n* = 1219	*P*-value†
**Child level**								
* P. falciparum* density (p/μl)								
No infection (0)								
Low (1–999)	−0.48 (−0.97, 0.02)		−0.03 (−2.07, 0.88)		−0.10 (−0.60, 0.37)		0.07 (−1.28, 1.16)	
Medium/high (≥1000)	0.23 (−0.51, 1.23)	0.151	−1.09 (−3.78, 2.95)	0.799	−0.15 (−0.84, 0.57)	0.876	0.51 (−1.18, 2.57)	0.866
Anaemia status								
Not anaemic								
Anaemic	−0.01 (−0.27, 0.25)	0.936	0.14 (−0.65, 0.86)	0.96	0.34 (0.00, 0.70)	0.053	−0.21 (−0.89, 0.47)	0.54
Sex								
Male								
Female	−0.08 (−0.33, 0.14)	0.524	−**1.08** (−**1.84,**−**0.18**)	**0.005**	0.03 (−0.28, 0.32)	0.841	0.09 (−0.64, 0.74)	0.8
Age (years)‡								
	**0.13** (**0.02, 0.25**)	**0.029**	−**0.56** (−**0.88,**−**0.27**)	**<0.001**	**0.26** (**0.13, 0.38**)	**<0.001**	−0.02 (−0.26, 0.19)	0.87
**Household-level**								
SES quintile								
Poorest								
Poor			−**0.19** (−**1.24, 0.92**)					
Median	–	–	**1.54** (**0.37, 2.74**)		–	–	–	–
Less poor			**1.07** (**0.01, 2.15**)	**<0.001**				
Least poor			**2.43** (**1.18, 3.68**)					
No. of children in household								
			−**0.24** (−**0.40,**−**0.08**)	**0.026**	–	–	–	–
Education of household head								
No schooling								
Primary	–	–	−**0.06** (−**0.81, 0.64**)		–	–	–	–
Secondary			**1.11** (−**0.35, 1.93**)	**0.012**				
College/degree			**3.33** (**1.10, 4.88**)					
Parent is literate								
No								
Yes	–	–	–	–	**0.49** (**0.18, 0.82**)	**0.003**	–	–
**School-level**								
Seating arrangement in classroom								
Desks or tables and chairs								
Floor	–	–	–	–	−**0.78** (−**1.34,**−**0.30**)	**0.005**	–	–
Division								
Diani								
Lunga Lunga	−**0.87** (−**1.34,**−**0.45**)						−**2.48** (−**3.85,**−**0.88**)	
Msambweni	**0.51** (−**0.31, 1.43**)	**<0.001**	–	–	–	–	−**1.69** (−**3.53,**−**0.00**)	**<0.001**
Kubo	−**0.47** (−**1.08, 0.22**)						−**3.76** (−**6.38,**−**1.94**)	

* Positive values indicate an increased score over reference group, and negative values indicate a decreased score over reference group (95% CI is the bias-corrected confidence interval).

† *P*-value is from multivariable Wald test derived from multivariable linear regression, bootstrapped and adjusted for school-level clustering.

‡ Modelled as a continuous variable.

The bold numbers refer to those with a *p*-value of 0.05 or less in the multivariable model.

A number of household factors were also associated with the scores. Higher household socio-economic status was associated with higher scores in the comprehension task in class 5 and the spelling in both classes. Lower scores were associated with living in a house with a high number of children for the class 5 comprehension task. Higher parental education levels were associated with higher scores in the class 5 comprehension and class 1 numeracy tasks. School environment and educational administrative zones were found to be associated with several of the tasks, with lower literacy and numeracy scores associated with children learning in classrooms without desks, and significantly higher spelling and numeracy scores in class 5 as well as attention and cognitive scores in class 1 found in children schooling in coastal Diani zone.

## Discussion

The evidence presented here shows that in this moderate malaria transmission setting, there is marked variation in the prevalence of *P. falciparum*, reaching 75% in some schools. There was also evidence that infection is strongly associated with anaemia, with the odds higher with increasing density of infection. The results also show potentially important variation in the malaria burden between the sexes and age groups and by school. The scale of observed health problems strongly supports the need for school health programmes aimed at reducing the health burden of malaria in school children. Despite this health burden, the analysis of educational data suggested no association between current health status and measurements of sustained attention and educational achievement.

The geographical heterogeneity observed in the prevalence of *P. falciparum* infection is likely to reflect a complexity of factors that influence vector distribution and density as well as vector–human contact and human infection (Greenwood 1989). The principal malaria vectors in the study are *Anopheles gambiae s.l.* and *An. funestus,* which in our study area have been shown to exhibit strong spatial and temporal heterogeneity related, in part, to variation in rainfall (Mbogo *et al.* 2003) and, more recently, variation in mosquito net use and type of household construction (Mutuku *et al.* 2011). Human–vector contact and human infection may also be influenced by proximity to vector breeding sites (Clarke *et al.* 2002; Pullan *et al.* 2010) and variation in personal protection measures (Snow *et al.* 1998) and net use (Pullan *et al.* 2010). Future geostatistical analysis will investigate the environmental correlates of the observed variation in infection patterns. Such geographical heterogeneity in infection risk has particular implications for the targeting of malaria interventions as well as for the possible impact of intervention (Kleinschmidt *et al.* 2007). School-level variation in the prevalence of anaemia may reflect the observed geographical variation in the prevalence of *P. falciparum* infection, but is also likely to be due to differences in food availability and the prevalence of helminth infection, important aetiological factors for anaemia.

The protective effect of sleeping under a mosquito net is consistent with previous cross-sectional findings (Noor *et al.* 2008; Pullan *et al.* 2010), whilst the strong association between *P. falciparum* infection and anaemia has been observed in other school-aged populations in East Africa (Stoltzfus *et al.* 1997; Olsen *et al.* 1998; Leenstra *et al.* 2004; Koukounari *et al.* 2008). The impact of chronic *P. falciparum* infection on haemoglobin levels is attributed to increased red blood cell destruction and decreased red blood cell production (Abdalla *et al.* 1980; Phillips & Pasvol 1992; Menendez *et al.* 2000), with high-density infections intensifying these processes. However, anaemia is multifactorial, and the findings of this study highlight additional contributory factors: stunting, indicative of poor nutritional intake for a sustained period during the childhood growth phase, was associated with increased odds of anaemia. This nutritional relationship is supported by the finding that at sea level, in the schools nearest the coast where the soil is infertile and the crop-growing potential is poor, the presence of a school feeding programme at the child’s school appears to be associated with a 50% decrease in odds of anaemia. Few studies to date have measured the effect of school feeding on anaemia (Jomaa *et al.* 2011), although provision of iron-fortified porridge and biscuits and cakes as part of school feeding programmes has been shown to be associated with a reduction in anaemia in Kenya, South Africa and Peru (Jacoby *et al.* 1998; van Stuijvenberg *et al.* 1999; Andang’o *et al.* 2007). Micronutrient deficiency is commonly found among school-aged children in malaria-endemic areas (Best *et al.* 2010), and infection with *P. falciparum* is bound to further increase the stress on the haemoglobin status in individuals who are already anaemic (Topley 1968; Nussenblatt & Semba 2002).

The lack of observed association between health status and sustained attention and education may not necessarily reflect an absence of effect of malaria on education. First, asymptomatic *P. falciparum* can persist for over three months and as children may be constantly re-infected, it is probable that infection has a cumulative effect on cognitive function over an extended period of time. Thus, the single time point of our cross-sectional design may not sufficiently capture the effects of recurrent, chronic infection over an extended period (Jukes *et al.* 2002; Thuilliez 2010). Second, the cross-sectional design meant that we were unable to capture information on past clinical attacks, which have previously been shown to be related to poor educational achievement (Fernando *et al.* 2003b). Third, malaria is just one of many contributing factors to poorer cognitive and educational performance, with socio-economic status and the educational environment of children’s homes playing an important role, as highlighted in the present study.

The association found in both classes between higher literacy and attention scores and indicators of SES is supported by previous findings where SES has been found to be strongly related to psychometric and education test scores in school children (Jukes *et al.* 2002). Increased SES is likely to be associated with increased stimulation, increased access to reading material and ownership of school-related materials, factors previously shown to be associated with increased academic achievement (Walker *et al.* 1998). This is supported by the fact that increased education of household heads and increased literacy were associated with improved performance in comprehension in class 5 and numeracy in class 1. As expected, there was a positive relationship between age and assessment scores for children in class 1. By contrast, increasing age was associated with lower scores in assessments in class 5. This seemingly contradictory observation could be attributed to the older children in class 5 having repeated earlier years because of poor educational performance, as is frequently seen in low-income countries (Heyneman & Loxley 1983; Fehrler *et al.* 2009). Also, poor children enrol in school later (Fentiman *et al.* 1999). The poorer scores in attention (class 1) and literacy (class 5) assessments observed in females are consistent with the recognized disparity between sexes in access to education and support in many low-income settings (Jukes *et al.* 2008). The strong variation in educational performance by administrative division is an indicator that there are aspects of the school divisional organization and management, such as the availability of books, the teacher–child contact time and the quality of teaching, which may influence educational outcomes (Heyneman & Loxley 1983; Boissiere 2004; Bhargava *et al.* 2005). The importance of the school environment is further demonstrated by the lower literacy and numeracy scores observed in class 1 children who learn in classrooms with no desks.

In conclusion, we have found a strong geographical variation in the prevalence of *P. falciparum* infection, underscoring the need for geographical targeting of malaria interventions. The observed strong association between infection and anaemia provides evidence of the, presumably cumulative, negative effects of asymptomatic *P. falciparum* infection on the haemoglobin status of school children. The ongoing trial of IST will provide an indication of how much of this effect can be reversed and whether malaria control can also improve the cognitive and educational performance of children in the current setting. The trial will also help understand how much of the observed poor performance is attributed to health factors and how much is attributed to teaching quality and household factors.

## Supporting Information

**Table S1** Characteristics of study children with health data only or health and education data (included in analysis) and study children with education data only (excluded from analysis).

**Table S2** Univariable analyses for associations of *Plasmodium falciparum* infection and anaemia and additional potential risk factors with a test of cognition (Ravens test), numeracy (Number Identification test) and a test of literacy (Spelling test) in class 1 children on the south coast of Kenya, 2010.

**Table S3.** Univariable analyses for associations of *Plasmodium falciparum* infection and anaemia and additional potential risk factors with a test of sustained attention (pencil tapping) in class 1 children on the south coast of Kenya, 2010.

**Table S4.** Univariable analyses for associations of *Plasmodium falciparum* infection and anaemia and additional potential risk factors with a test of cognition (Silly Sentences), numeracy (Written Numeracy test), literacy (Spelling test) and sustained attention (Code Transmission test) in class 5 children on the south coast of Kenya, 2010.
