# Supplementary material for: Plasmodium falciparum, anaemia and cognitive and educational performance among school children in an area of moderate malaria transmission: baseline results of a cluster randomized trial on the coast of Kenya
Source: Trop Med Int Health. 2012 Apr 19;17(5):532–49. doi: 10.1111/j.1365-3156.2012.02971.x (PMC3506732; doi:10.1111/j.1365-3156.2012.02971.x)
Supplement: Supplementary file 1 [file tmi0017-0532-SD1.docx]

**Additional File 2:** Characteristics of study children with health data only or health and education data (included in analysis) and study children with education data only (excluded from analysis).

| **Variable** | **Children with health data (included in this analysis) N (%)^1,^ n=2400** | **Children with only education data (excluded from analysis) N (%)^1^  n=275** |
| --- | --- | --- |
| **Division^2^** |  |  |
| Diani  Lunga Lunga  Msambweni  Kubo | 636 (26.5)  960 (40.0)  312 (13.0)  492 (20.5) | 51 (18.6)  128 (46.5)  60 (21.8)  36 (13.1) |
| **Sex** |  |  |
| Male  Female | 1167 (48.6)  1233 (51.4) | 132 (48.0)  143 (52.0) |
| **Age (yrs)** |  |  |
|  | 10.34 (2.81) | 10.31 (2.97) |
| **Age groups (yrs)** |  |  |
| 5-10  11-12  13-18 | 940 (39.2)  860 (34.6)  630 (26.2) | 111 (40.4)  87 (31.6)  77 (28.0) |
| **Education level of household head** |  |  |
| No schooling  Primary  Secondary  College/degree | 814 (34.3)  1228 (51.8)  255 (10.8)  74 (3.1) | 95 (35.9)  141 (53.2)  22 (8.3)  7 (2.6) |
| **Number of people in household** |  |  |
|  | 7.06 (2.52) | 7.11 (2.34) |
| **Number of children in household** |  |  |
|  | 4.82 (2.18) | 4.76 (2.11) |
| **SES quintile** |  |  |
| Poorest  Poor  Median  Less poor  Least poor | 577 (24.2)  504 (21.1)  423 (17.7)  459 (19.3)  422 (17.7) | 74 (27.9)  54 (20.4)  63 (23.8)  41 (15.5)  33 (12.4) |
| **Child sleeps under a net** |  |  |
| No  Yes | 880 (37.2)  1489 (62.8) | 87 (33.0)  177 (67.0) |
| **Child been dewormed in last year** |  |  |
| No  Yes | 442 (18.6)  1824 (77.0) | 59 (23.4)  193 (76.6) |
| **Malaria control activities in school^2^** |  |  |
| No  Yes | 1814 (74.1)  586 (25.9) | 224 (81.5)  51 (18.5) |
| **School feeding programme in school^2^** |  |  |
| No  Yes | 1115 (46.5)  1285 (53.5) | 143 (52.0)  132 (48.0) |

^1^Displayed as number and percentage except for continuous variables, displayed as Mean and Standard Deviation (SD).

^2^ Measured at the school level.
