# Supplementary material for: Plasmodium falciparum, anaemia and cognitive and educational performance among school children in an area of moderate malaria transmission: baseline results of a cluster randomized trial on the coast of Kenya
Source: Trop Med Int Health. 2012 Apr 19;17(5):532–49. doi: 10.1111/j.1365-3156.2012.02971.x (PMC3506732; doi:10.1111/j.1365-3156.2012.02971.x)
Supplement: Supplementary file 2 [file tmi0017-0532-SD2.docx]

**Table A1:** Univariable analyses for associations of *P. falciparum* infection and anaemia and additional potential risk factors with a test of cognition (Ravens test), numeracy (Number Identification test) and a test of literacy (Spelling test) in class 1 children on the South Coast of Kenya, 2010.

|  | | **RAVENS COGNITIVE ( non verbal reasoning) TEST class 1** | | |  | **NUMBER IDENTIFICATION TEST class 1** | | |  | **SPELLING TEST class 1** | | |
| --- | --- | --- | --- | --- | --- | --- | --- | --- | --- | --- | --- | --- |
| Risk factor | Number of children  n (%)^1,2^  1135 | Mean score^3^  (0-20) (SD) | Mean difference between test performance  (95% CI) | P value^4^ |  | Mean score^3^  (0-20) (SD) | Mean difference between test performance  (95% CI) | P value^4^ |  | Mean score^3^  (0-20) (SD) | Mean difference between test performance  (95% CI) | P value^4^ |
| **CHILD LEVEL** |  |  |  |  |  |  |  |  |  |  |  |  |
| **Sex**  Male  Female | 572 (50.4)  563 (49.6) | 7.45 (2.66)  7.39 (2.50) | -0.06 (-0.33, 0.19) | 0.679 |  | 3.24 (2.90)  3.14 (2.60) | -0.10 (-0.45, 0.19) | 0.542 |  | 7.51 (4.27)  7.68 (4.57) | 0.17 (-0.41, 0.80) | 0.578 |
| **Age (years)**^5^ | 12.56 (1.55) | 7.42 (2.58) | 0.09 (-0.01, -0.20) | 0.106 |  | 3.19 (2.75) | 0.25 (0.12, 0.37) | <0.001 |  | 7.59 (4.42) | 0.23 (0.02, 0.44) | 0.030 |
| ***P.falciparum* density (p/µl)**  No infection (0)  Low (1-999)  Medium/High (≥1000) | 949 (83.6)  136 (12.0)  50 (4.4) | 7.43 (2.57)  7.17 (2.50)  7.86 (3.03) | -0.26 (-0.70, 0.18)  0.43 (-0.42, 1.43) | 0.365 |  | 3.18 (2.78)  3.29 (2.77)  3.14 (2.29) | 0.12 (-0.46, 0.67)  -0.04 (-0.72, 0.67) | 0.901 |  | 7.45 (4.42)  8.22 (4.64)  8.52 (3.52) | 0.77 (-0.54, 2.20)  1.07 (-0.12, 2.34) | 0.179 |
| **Anaemia status**  Not anaemic  Anaemic | 596 (52.5)  539 (47.5) | 7.38 (2.57)  7.46 (2.59) | 0.08 (-0.21, 0.36) | 0.564 |  | 3.06 (2.61)  3.33 (2.90) | 0.27 (-0.02, 0.64) | 0.105 |  | 7.44 (4.45)  7.76 (4.39) | 0.32 (-0.28, 0.97) | 0.324 |
| **WAZ (z scores)** ^2^  Not wasted  Wasted | 683 (75.6)  221 (24.4) | 7.35 (2.47)  7.63 (2.94) | 0.28 (-0.15, 0.75) | 0.221 |  | 3.00 (2.62)  3.20 (2.72) | 0.20 (-0.26, 0.59) | 0.357 |  | 7.48 (4.48)  7.63 (4.58) | 0.15 (-0.73, 0.95) | 0.733 |
| **HAZ (z scores)**  Not stunted  Stunted | 850 (75.0)  283 (25.0) | 7.39 (2.52)  7.53 (2.75) | 0.15 (-0.27, 0.59) | 0.494 |  | 3.19 (2.75)  3.22 (2.77) | 0.03 (-0.39, 0.42) | 0.884 |  | 7.62 (4.51)  7.51 (4.15) | -0.12 (-0.74, 0.47) | 0.723 |
| **BMIZ (z scores)**  Not thin  Thin | 923 (81.5)  209 (18.5) | 7.47 (2.57)  7.22 (2.62) | -0.25 (-0.64, 0.10) | 0.164 |  | 3.20 (2.78)  3.17 (2.64) | -0.03 (-0.38, 0.38) | 0.880 |  | 7.67 (4.40)  7.20 (4.53) | -0.48 (-1.14, 0.29) | 0.203 |
| **Child been dewormed in last year**  No  Yes | 277 (25.8)  796 (74.2) | 7.49 (2.60)  7.41 (2.53) | -0.08 (-0.49, 0.38) | 0.716 |  | 3.24 (2.87)  3.23 (2.76) | -0.01 (-0.47, 0.44) | 0.964 |  | 7.62 (4.14)  7.66 (4.52) | 0.05 (-0.76, 0.78) | 0.905 |
| **Child missed school previous week**^2^  No  Yes | 651 (62.4)  393 (37.6) | 7.35 (2.60)  7.51 (2.50) | 0.16 (-0.12, 0.46) | 0.296 |  | 3.07 (2.71)  3.42 (2.84) | 0.36 (-0.02, 0.74) | 0.063 |  | 7.40 (4.37)  7.89 (4.53) | 0.49 (-0.01, 1.04) | 0.070 |
| **Child ate breakfast before assessed**  No  Yes | 331 (39.5)  791 (70.5) | 7.12 (2.26)  7.53 (2.68) | 0.41 (0.07, 0.73) | 0.015 |  | 3.01 (2.67)  3.24 (2.78) | 0.23 (-0.19, 0.63) | 0.256 |  | 7.33 (4.23)  7.69 (4.51) | 0.35 (-0.31, 1.00) | 0.282 |
| **Child failed a grade**^2^  No  Yes | 719 (66.3)  365 (33.7) | 7.40 (2.54)  7.32 (2.52) | -0.08 (-0.37, 0.27) | 0.642 |  | 3.09 (2.66)  2.50 (2.97) | 0.40 (0.05, 0.80) | 0.038 |  | 7.54 (4.52)  7.82 (4.25) | 0.27 (-0.36, 0.85) | 0.367 |
| **Family has books at home**  No  Yes | 788 (71.4)  315 (28.6) | 7.39 (2.58)  7.30 (2.59) | -0.18 (-0.60, 0.17) | 0.373 |  | 3.11 (2.69)  3.39 (2.98) | 0.28 (-0.08, 0.67) | 0.149 |  | 7.44 (4.41)  7.95 (4.51) | 0.51 (-0.19, 1.20) | 0.145 |
| **HOUSEHOLD LEVEL** |  |  |  |  |  |  |  |  |  |  |  |  |
| **Education Level of household head**  No schooling  Primary  Secondary  College/degree | 388 (34.5)  593 (52.8)  103 (9.2)  39 (3.5) | 7.23 (2.41)  7.45 (2.65)  7.92 (2.83)  7.64 (2.35) | 0.22 (-0.08, 0.57)  0.69 (0.05, 1.37)  0.41 (-0.25, 1.16) | 0.130 |  | 2.98 (2.62)  3.21 (2.63)  3.74 (3.11)  3.77 (4.40) | 0.23 (-0.14, 0.55)  0.76 (-0.02, 1.46)  0.79 (-0.60, 2.39) | 0.167 |  | 7.29 (4.18)  7.66 (4.41)  8.06 (5.04)  7.95 (5.35) | 0.37 (-0.26, 1.00)  0.77 (-0.46, 1.97)  0.65 (-1.08, 2.55) | 0.611 |
| **Child sleeps under a net**  No  Yes | 373 (33.4)  745 (66.6) | 7.48 (2.75)  7.35 (2.45) | -0.13 (-0.45, 0.23) | 0.432 |  | 3.17 (2.69)  3.21 (2.81) | 0.04 (-0.27, 0.37) | 0.794 |  | 7.51 (4.17)  7.65 (4.54) | 0.13 (-0.49, 0.77) | 0.684 |
| **SES quintiles**  Poorest  Poor  Median  Less poor  Least poor | 287 (25.4)  256 (22.6)  196 (17.3)  203 (18.0)  189 (16.7) | 7.36 (2.46)  7.38 (2.52)  7.37 (2.73)  7.45 (2.46)  7.61 (2.83) | 0.02 (-0.36, 0.35)  0.00 (-0.53, 0.58)  0.09 (-0.32, 0.52)  0.25 (-0.28, 0.71) | 0.860 |  | 3.03 (2.76)  3.16 (2.66)  3.06 (2.73)  3.17 (2.79)  3.74 (2.86) | 0.13 (-0.39, 0.59)  0.02 (-0.52, 0.57)  0.14 (-0.48, 0.81)  0.61 (-0.01, 1.22) | 0.202 |  | 7.04 (4.34)  7.24 (4.28)  7.50 (4.07)  8.11 (4.34)  8.43 (4.97) | 0.21 (-0.48, 1.01)  0.46 (-0.39, 1.34)  1.07 (0.22, 2.06)  1.39 (0.44, 2.44) | 0.052 |
| **Household size**^5^ | 7.20 (2.60) | 7.40 (2.56) | -0.07 (-0.14, -0.01) | 0.041 |  | 3.20 (2.77) | 0.01 (-0.06, 0.08) | 0.859 |  | 7.60 (4.42) | 0.04 (-0.07, 0.16) | 0.515 |
| **Number of children in house**^5^ | 5.06 (2.25) | 7.40 (2.56) | -0.07 (-0.14, -0.01) | 0.045 |  | 3.20 (2.77) | 0.00 (-0.09, 0.10) | 0.932 |  | 7.60 (4.42) | 0.09 (-0.05, 0.22) | 0.193 |
| **Parent is literate**  No  Yes | 398 (35.5)  722 (64.5) | 7.27 (2.38)  7.51 (2.69) | 0.24 (-0.08, 0.56) | 0.154 |  | 2.92 (2.52)  3.34 (2.87) | 0.41 (0.087, 0.76) | 0.021 |  | 7.15 (4.15)  7.83 (4.56) | 0.68 (-0.01, 1.33) | 0.050 |
| **Language parents speak with child**  Mothertongue  English/Swahili | 925 (82.8)  192 (17.2) | 7.47 (2.61)  7.21 (2.56) | -0.26 (-0.72, 0.23) | 0.265 |  | 3.16 (2.77)  3.33 (2.69) | 0.17 (-0.41, 0.87) | 0.594 |  | 7.68 (4.38)  7.07 (4.68) | -0.61 (-1.90, 0.99) | 0.434 |
| **SCHOOL LEVEL** |  |  |  |  |  |  |  |  |  |  |  |  |
| **Child teacher ratio**  15-34  35-44  45-54  55-64  ≥65 | 187 (16.5)  299 (26.3)  350 (30.8)  120 (10.6)  179 (15.8) | 7.66 (2.54)  7.40 (2.40)  7.72 (3.05)  6.78 (2.23)  7.03 (1.96) | -0.26 (-0.80, 0.31)  0.07 (-0.58, 0.68)  -0.88 (-1.35, -0.38)  -0.62 (-1.32, -0.06) | 0.003 |  | 3.34 (2.61)  2.95 (2.33)  3.82 (3.16)  2.5 0 (2.70)  2.65 (2.49) | -0.38 (-1.42, 0.66)  0.48 (-0.61, 1.54)  -0.84 (-2.08, 0.30)  -0.69 (-1.76, 0.22) | 0.063 |  | 7.61 (4.58)  7.43 (4.58)  9.01 (4.52)  5.98 (3.29)  6.15 (3.50) | -0.18 (-2.33, 2.42)  1.40 (-0.53, 3.84)  -1.64 (-3.69, 0.75)  -1.46 (-3.19, 0.69) | <0.001 |
| **Seating arrangement in classroom**  Desks or tables and chairs  Floor | 967 (85.2)  168 (14.8) | 7.36 (2.57)  7.76 (2.65) | 0.40 (-0.41, 1.21) | 0.338 |  | 3.32 (2.87)  2.46 (1.84) | -0.85 (-1.40, -0.25) | 0.003 |  | 7.86 (4.46)  6.00 (3.80) | -1.87 (-3.70, -0.57) | 0.022 |
| **School malaria control activities**  No  Yes | 867 (76.4)  268 (23.6) | 7.39 (2.55)  7.52 (2.69) | 0.14 (-0.38, 0.64) | 0.519 |  | 3.18 (2.72)  3.22 (2.86) | 0.04 (-0.67, 0.74) | 0.915 |  | 7.49 (4.48)  7.92 (4.22) | 0.42 (-1.05, 1.83) | 0.576 |
| **School feeding programme**  No  Yes | 525 (46.3)  610 (53.7) | 7.56 (2.72)  7.30 (2.46) | -0.27 (-0.70, 0.16) | 0.240 |  | 3.27 (2.56)  2.12 (2.91) | -0.14 (-0.74, 0.53) | 0.661 |  | 7.61 (4.25)  7.57 (4.56) | -0.04 (-1.41, 1.33) | 0.952 |
| **Administrative Division**  Diani  Lunga Lunga  Msambweni  Kubo | 303 (26.7)  457 (40.3)  139 (12.2)  236 (20.8) | 7.79 (2.72)  6.98 (2.47)  8.22 (2.79)  7.32 (2.32) | -0.81 (-1.28, -0.40)  -0.43 (-0.34, 1.33)  -0.47 (-1.08, 0.13) | >0.001 |  | 3.63 (2.58)  3.06 (3.00)  2.87 (2.31)  3.08 (2.66) | -0.57 (-1.21, 0.11)  -0.76 (-1.55, -0.03)  -0.55 (-1.56, 0.79) | 0.141 |  | 8.99 (4.38)  7.20 (4.03)  6.79 (4.89)  7.03 (4.54) | -1.79 (-3.28, -0.23)  -2.19 (-4.82, 0.50)  -1.95 (-3.93, 0.42) | 0.080 |

^1^ 1135 observations included for Ravens test. 1134 observations included for number identification. 1131 observations included for spelling test. Percentage children per characteristic shown for 1135 children.

^2^All missing <3% with the exception of WAZ-20.3%, children missed school previous week-8.0%, child failed a grade-4.5%

^3^Positive values indicate an increased score over reference group and negative values indicate a decreased score over reference group (95% CI is the bias corrected confidence interval)

^4^ P value is from multivariable Wald test derived from multivariable linear regression, bootstrapped and adjusted for school level clustering
