# Supplementary material for: Plasmodium falciparum, anaemia and cognitive and educational performance among school children in an area of moderate malaria transmission: baseline results of a cluster randomized trial on the coast of Kenya
Source: Trop Med Int Health. 2012 Apr 19;17(5):532–49. doi: 10.1111/j.1365-3156.2012.02971.x (PMC3506732; doi:10.1111/j.1365-3156.2012.02971.x)
Supplement: Supplementary file 3 [file tmi0017-0532-SD3.docx]

**Table A2:** Univariable analyses for associations of *P. falciparum* infection and anaemia and additional potential risk factors with a test of sustained attention (pencil tapping) in class 1 children on the South Coast of Kenya, 2010.

|  | **PENCIL TAP ATTENTION TEST class 1** | | | | | | |
| --- | --- | --- | --- | --- | --- | --- | --- |
|  | **Probability of children engaging in the task** | | | | **Scores if children are engaged** | | |
| Risk factor | Number of children  (%)^1,2^  n=1135 | Number of children engaged in task (%)^3^  n= 998 | OR of engagement;  (95% CI) | P- value^4^ | Mean score if engaged  (1-20) (SD) | Mean difference between test performance^5^  (95% CI) | P value^6^ |
| **CHILD LEVEL** |  |  |  |  |  |  |  |
| **Sex**  Male  Female | 572 (50.4)  563 (49.6) | 513 (89.7)  485 (86.2) | 1  0.71 (0.49, 1.02) | 0.064 | 14.17 (4.99)  13.36 (5.11) | -0.80 (-1.35, 0.21) | 0.006 |
| **Age (years)** | 12.56 (1.55) | 12.56 (1.55) | 1.20 (1.07, 1.35) | 0.002 | 13.78 (5.07) | 0.57 (0.36, 0.74) | <0.001 |
| ***P.falciparum* density (p/µl)**  No infection (0)  Low (1-999)  Medium/High (≥1000) | 949 (83.6)  136 (12.0)  50 (4.4) | 929 (87.4)  121 (89.0)  48 (96.0) | 1  1.12 (0.61, 2.04)  3.48 (0.82, 14.82) | 0.131 | 13.71 (5.07)  14.13 (4.91)  14.13 (5.31) | 0.42 (-0.53, 1.53)  0.42 (-0.82, 1.58) | 0.564 |
| **Anaemia status**  Not anaemic  Anaemic | 596 (52.5)  539 (47.5) | 515 (86.4)  483 (89.6) | 1  1.28 (0.88, 1.87) | 0.198 | 13.63 (5.04)  13.94 (5.08) | 0.31 (-0.28, 0.90) | 0.290 |
| **WAZ (z scores)**  Not wasted  Wasted | 683 (75.6)  221 (24.4) | 597 (87.4)  188 (85.1) | 1  0.80 (0.51, 1.26) | 0.341 | 13.55 (5.08)  13.62 (5.06) | -0.09 (-0.85, 0.65) | 0.891 |
| **HAZ (z scores)**  Not stunted  Stunted | 850 (75.0)  283 (25.0) | 742 (87.3)  254 (89.8) | 1  1.29 (0.83-2.02) | 0.249 | 13.68 (5.01)  14.07 (5.22) | 0.39 (-0.37, 1.06) | 0.278 |
| **BMIZ (z scores)**  Not thin  Thin | 923 (81.5)  209 (18.5) | 816 (88.4)  179 (85.7) | 1  0.82 (0.52, 1.29) | 0.394 | 13.79 (5.00)  13.70 (5.40) | -0.09 (-0.85, 0.65) | 0.820 |
| **Child been dewormed in last year**  No  Yes | 277 (25.8)  796 (74.2) | 241 (87.0)  704 (88.4) | 1  1.19 (0.77, 1.83) | 0.430 | 14.27 (4.86)  13.75 (5.04) | -0.52 (-1.29, 0.29) | 0.205 |
| **Child missed schl in previous week**  No  Yes | 651 (62.4)  393 (37.6) | 564 (86.6)  351 (89.3) | 1  1.28 (0.86, 1.92) | 0.220 | 13.84 (5.00)  13.85 (5.13) | 0.00 (-0.57, 0.61) | 0.990 |
| **Child ate breakfast on day of test**  No  Yes | 331 (29.5)  791 (70.5) | 307 (92.8)  680 (89.0) | 1  0.46 (0.29, 0.75) | <0.001 | 13.63 (5.00)  13.81 (5.13) | 0.17 (-0.52, 0.86) | 0.627 |
| **Child failed a grade**  No  Yes | 719 (66.3)  365 (33.7) | 635 (88.3)  320 (87.7) | 1  0.92 (0.62, 1.37) | 0.688 | 13.55 (5.11)  14.25 (4.96) | 0.71 (0.00, 1.45) | 0.062 |
| **HOUSEHOLD LEVEL** |  |  |  |  |  |  |  |
| **Education Level of household head**  No schooling  Primary  Secondary  College/degree | 388 (34.5)  593 (52.8)  103 (9.2)  39 (3.5) | 338 (87.1)  523 (88.2)  93 (90.3)  36 (92.3) | 1  1.18 (0.78, 1.77)  1.65 (0.78, 3.51)  2.08 (0.60, 7.28) | 0.405 | 14.25 (4.96)  13.70 (5.06)  12.69 (5.09)  13.33 (5.84) | -0.55 (-0.27, 1.46)  -1.57 (-2.90, -0.18)  -0.92 (-2.93, 0.87) | 0.110 |
| **Child sleeps under a net**  No  Yes | 373 (33.4)  745 (66.6) | 332 (89.0)  654 (87.8) | 1  0.95 (0.63, 1.43) | 0.796 | 14.42 (5.00)  13.47 (5.08) | -0.96 (-1.73, -0.23) | 0.013 |
| **SES quintiles**  Poorest  Poor  Median  Less poor  Least poor | 287 (25.4)  256 (22.6)  196 (17.3)  203 (18.0)  189 (16.7) | 257 (89.6)  227 (88.7)  174 (88.8)  177 (87.2)  162 (85.7) | 1  0.94 (0.54, 1.65)  1.01 (0.55, 1.86)  0.86 (0.48, 1.54)  0.73 (0.41, 1.31) | 0.830 | 14.48 (5.04)  14.27 (4.99)  13.43 (4.97)  13.16 (5.20)  13.02 (5.01) | -0.21 (-1.23, 0.69)  -1.05 (-2.04, -0.17)  -1.31 (-2.42, -0.38)  -1.46 (-2.53, -0.40) | 0.002 |
| **Household size** | 7.20 (2.60) | 7.20 (2.60) | 1.06 (0.98, 1.16) | 0.134 | 13.78 (5.07) | 0.18 (0.06, 0.32) | 0.007 |
| **Number of children in house** | 5.06 (2.25) | 5.06 (2.25) | 1.09 (0.99, 1.21) | 0.068 | 13.78 (5.07) | 0.06 (0.08, 0.19) | 0.428 |
| **Parent is literate**  No  Yes | 398 (35.5)  722 (64.5) | 348 (87.4)  639 (88.5) | 1  1.18 (0.79, 1.74) | 0.421 | 14.11 (5.02)  13.61 (5.08) | -0.50 (-1.12, 0.13) | 0.122 |
| **Language parents speak with child**  Mother tongue  English/Swahili | 925 (82.8)  192 (17.2) | 818 (88.4)  167 (87.0) | 1  0.88 (0.53, 1.44) | 0.605 | 13.96 (4.94)  12.79 (5.56) | -1.17 (-2.09, -0.18) | 0.017 |
| **Family has books at home**  No  Yes | 788 (71.4)  315 (28.6) | 692 (87.8)  279 (88.6) | 1  1.16 (0.76, 1.76) | 0.481 | 13.98 (4.95)  13.25 (5.29) | -0.72 (-1.43, -0.09) | 0.042 |
| **SCHOOL LEVEL** |  |  |  |  |  |  |  |
| **Child teacher ratio**  15-34  35-44  45-54  55-64  ≥65 | 187 (16.5)  299 (26.3)  350 (30.8)  120 (10.6)  179 (15.8) | 167 (89.3)  257 (86.0)  308 (88.0)  109 (90.8)  157 (87.7) | 1  0.73 (0.35, 0.51)  0.86 (0.42, 1.75)  1.18 (0.45, 3.08)  0.83 (0.36, 1.88) | 0.821 | 13.87 (5.04)  13.45 (5.02)  13.80 (5.12)  13.80 (5.26)  14.16 (4.94) | -0.42 (-1.82, 1.05)  -0.08 (-1.48, 1.50)  -0.08 (-1.47, 1.77)  -0.28 (-1.19, 1.94) | 0.862 |
| **Seating arrangement in classroom**  Desks or tables and chairs  Floor | 967 (85.2)  168 (14.8) | 848 (87.7)  150 (89.3) | 1  1.13 (0.58, 2.20) | 0.709 | 13.77 (5.09)  13.82 (4.92) | 0.05 (-0.70, 1.12) | 0.914 |
| **School malaria control activities**  No  Yes | 867 (76.4)  268 (23.6) | 757 (87.3)  241 (89.9) | 1  1.30 (0.74, 2.29) | 0.351 | 13.57 (5.07)  14.43 (5.00) | 0.86 (-0.21, 1.73) | 0.089 |
| **School feeding programme**  No  Yes | 525 (46.3)  610 (53.7) | 475 (90.5)  523 (85.7) | 1  0.63 (0.40, 0.99) | 0.049 | 14.00 (5.03)  13.58 (5.09) | -0.42 (-1.21, 0.36) | 0.321 |
| **Administrative Division**  Diani  Lunga Lunga  Msambweni  Kubo | 303 (26.7)  457 (40.3)  139 (12.2)  236 (20.8) | 271 (89.4)  415 (90.8)  120 (86.3)  192 (81.4) | 1  1.17 (0.67, 2.03)  0.74 (0.37, 1.49) 0.51 (0.29, 0.92) | 0.032 | 14.16 (5.06)  14.35 (4.95)  12.90 (5.19)  12.56 (4.98) | 0.19 (-0.70, 1.15)  -1.26 (-2.44, -0.19)  -0.60 (-2.87, -0.39) | <0.001 |

^1^ 1135 observations included for Pencil-tap attention test. Displayed as number and percentage except for continuous variables, displayed as mean and standard deviation (SD).

^2^All missing <3% with the exception of WAZ-20.3%, children missed school previous week-8.0%, child failed a grade-4.5%

^3^Only children found to be engaged in task are included.

^4^P value is from likelihood ratio test comparing multilevel logistic regression models (adjusting for school level clustering), with and without character of interest.

^5^Positive values indicate an increased score over reference group and negative values indicate a decreased score over reference group (95% CI is the bias corrected confidence interval)

^6^ P-value is from multivariable Wald test derived from multivariable linear regression, bootstrapped and adjusted for school level clustering
