# Supplementary material for: Plasmodium falciparum, anaemia and cognitive and educational performance among school children in an area of moderate malaria transmission: baseline results of a cluster randomized trial on the coast of Kenya
Source: Trop Med Int Health. 2012 Apr 19;17(5):532–49. doi: 10.1111/j.1365-3156.2012.02971.x (PMC3506732; doi:10.1111/j.1365-3156.2012.02971.x)
Supplement: Supplementary file 4 [file tmi0017-0532-SD4.docx]

**Table A3:** Univariable analyses for associations of *P. falciparum* infection and anaemia and additional potential risk factors with a test of cognition (Silly Sentences), numeracy (Written Numeracy test), literacy (Spelling test) and sustained attention (Code Transmission test) in class 5 children on the south coast of Kenya, 2010.

|  | | **SILLY SENTENCES COMPREHENSION TEST class 5** | | | | **WRITTEN NUMERACY TEST class 5** | | | **SPELLING TEST class 5** | | | **CODE TRANSMISSION TEST class 5** | | |
| --- | --- | --- | --- | --- | --- | --- | --- | --- | --- | --- | --- | --- | --- | --- |
| **Risk factor** | Number of children  N (%)^1,2^  1229 | | Mean score^3^  (0-40) (SD) | Mean difference between test performance  (95% CI) | P value^4^ | Mean score^3^  (0-38) (SD) | Mean difference between test performance  (95% CI) | P value^4^ | Mean score^3^  (0-20) (SD) | Mean difference between test performance  (95% CI) | P value^4^ | Mean score^3^  (0-20) (SD) | Mean difference between test performance  (95% CI) | P value^4^ |
| **CHILD LEVEL** |  | |  |  |  |  |  |  |  |  |  |  |  |  |
| **Sex**  Male  Female | 578 (47.0)  651 (53.0) | | 29.33 (6.30)  28.56 (6.48) | -0.77 (-1.51, 0.03) | 0.052 | 28.56 (5.81)  28.59 (5.74) | 0.04 (-0.66, 0.73) | 0.917 | 22.54 (8.48)  21.69 (7.93) | -0.86 (-1.67, 0.07) | 0.051 | 10.69 (5.52)  10.19 (5.73) | -0.50 (-1.20, 0.15) | 0.166 |
| **Age (years)**^5^ | 12.58 (1.52) | | 28.93 (6.40) | -0.70 (-1.03,-0.44) | 0.039 | 28.58 (5.77) | -0.04 (-0.27, 0.17) | 0.732 | 22.09 (8.20) | -1.37 (-1.72,-1.04) | <0.001 | 10.43 (5.64) | -0.26 (-0.44,-0.07) | 0.008 |
| ***P.falciparum* density (p/µl)**  No infection (0)  Low (1-999)  High (1000>) | 1106 (90.0)  101 (8.2)  22 (1.8) | | 28.94 (6.44) 28.94 (5.97)  28.18 (6.75) | 0.00 (-1.63, 1.42  -0.76 (-3.60, 2.77) | 0.890 | 28.51 (5.86)  29.25 (5.14)  29.00 (3.82) | 0.74 (-0.47, 2.06)  0.49 (-1.21, 2.61) | 0.474 | 22.12 (8.27)  22.16 (7.44)  20.36 (8.17) | 0.04 (-1.97, 2.28)  -1.75 (-6.11, 2.46) | 0.725 | 10.43 (5.63)  10.57 (5.84)  9.59 (5.15) | 0.15 (-1.32, 1.68)  -0.84 (-3.16, 1.27) | 0.829 |
| **Anaemia status**  Not anaemic  Anaemic | 696 (56.6)  533 (43.4) | | 28.97 (6.50)  28.86 (6.28) | -0.11 (-0.92, 0.71) | 0.793 | 28.57 (5.92)  28.59 (4.80) | 0.02 (-0.74, 0.77) | 0.949 | 21.94 (8.54)  22.28 (7.75) | 0.34 (-0.76, 1.45) | 0.528 | 10.31 (5.66)  10.58 (5.61) | 0.27 (-0.33, 0.84) | 0.360 |
| **HAZ (z scores)**  Not stunted  Stunted | 916 (74.7)  311 (25.3) | | 29.09 (6.55)  28.40 (5.94) | -0.69 (-1.42, 0.06) | 0.070 | 28.80 (5.66)  27.92 (6.06) | -0.88 (-1.76,-0.03) | 0.042 | 22.28 (8.20)  21.51 (8.21) | -0.77 (-2.00, 0.33) | 0.202 | 10.35 (5.71)  10.64 (5.43) | 0.29 (-0.38, 0.96) | 0.410 |
| **BMIZ (z scores)**  Not thin  Thin | 1000 (81.5)  227 (18.5) | | 28.80 (6.47)  29.44 (6.11) | 0.65 (-0.22, 1.60) | 0.164 | 28.51 (5.83)  28.85 (5.55) | 0.34 (-0.39, 1.14) | 0.400 | 21.83 (8.26)  23.21 (7.90) | 1.38 (0.26, 2.49) | 0.016 | 10.35 (5.63)  10.77 (5.69) | 0.43 (-0.41, 1.24) | 0.302 |
| **HOUSEHOLD LEVEL** |  | |  |  |  |  |  |  |  |  |  |  |  |  |
| **Education of household head**  No schooling  Primary  Secondary  College/degree | 415 (34.2)  614 (50.6)  150 (12.4)  34 (2.8) | | 28.20 (6.10)  28.74 (6.47)  30.45 (6.64)  33.24 (5.33) | 0.55 (-0.19, 1.28)  2.25 (1.03, 3.46)  5.04 (3.06, 6.74) | <0.001 | 28.23 (5.84)  28.57 (5.89)  29.36 (5.13)  29.12 (5.69) | 0.34 (-0.25, 0.99)  1.13 (0.31, 2.19)  0.88 (-1.03, 3.10) | 0.207 | 21.18 (7.80)  21.95 (8.24)  23.84 (8.39)  25.76 (9.39) | 0.77 (-0.33, 1.65)  2.66 (1.38, 4.00)  4.58 (0.93, 8.49) | <0.001 | 9.91 (4.48)  10.64 (5.75)  10.75 (5.61)  11.06 (5.43) | 0.74 (-0.27, 1.46)  0.83 (-0.38, 2.13)  1.15 (-0.90, 2.96) | 0.232 |
| **Child sleeps under a net**  No  Yes | 494 (40.6)  722 (59.4) | | 28.39 (6.51)  29.28 (6.33) | 0.88 (0.02, 1.80) | 0.059 | 28.83 (5.65)  28.39 (5.85) | -0.44 (-1.14, 0.30) | 0.214 | 21.41 (8.27)  22.52 (8.16) | 1.12 (0.17, 2.13) | 0.027 | 10.19 (5.83)  10. 57 (5.51) | 0.37 (-0.31, 0.97) | 0.253 |
| **Child been dewormed in last year**  No  Yes | 155 (13.4)  1003 (86.6) | | 28.01 (6.75)  29.12 (6.33) | 1.11 (-0.46, 2.59) | 0.166 | 27.66 (6.43)  28.72 (5.68) | 1.06 (-0.59, 2.89) | 0.231 | 21.26 (8.63)  22.21 (8.13) | 0.95 (-1.23, 3.10) | 0.397 | 9.81 (5.64)  10.52 (5.61) | 0.72 (-0.39, 1.78) | 0.186 |
| **SES quintile**  Poorest  Poor  Median  Less poor  Least poor | 283 (23.2)  240 (19.7)  222 (18.2)  246 (20.2)  228 (18.7) | | 27.64 (6.38)  27.67 (6.58)  29.30 (6.29)  29.24 (6.18)  31.10 (5.99) | 0.03 (-0.98, 1.19)  1.66 (0.49, 2.83)  1.61 (0.60, 2.71)  3.46 (2.30, 4.70)` | <0.001 | 27.99 (5.86)  28.30 (5.88)  28.70 (6.06)  28.77 (6.11)  29.28 (4.75) | 0.31 (-0.59, 1.57)  0.71 (-0.17, 1.66)  0.78 (-0.32, 1.93)  1.29 (0.34, 2.40)` | 0.057 | 20.04 (8.28)  20.40 (8.33)  22.68 (7.45)  22.38 (8.23)  25.40 (7.53) | 0.36 (-1.03, 1.70)  2.64 (1.48, 4.08)  2.34 (1.08, 3.65)  5.36 (3.70, 7.06) | <0.001 | 9.90 (5.50)  10.44 (5.69)  9.98 (5.60)  11.06 (5.77)  10.80 (5.61) | 0.53 (-0.25, 1.49)  0.07 (-0.96, 1.20)  1.16 (0.38, 2.15)  0.89 (0.13, 1.84) | 0.064 |
| **Household size**^5^ | 7.21 (2.61) | | 28.92 (6.42) | -0.22 (-0.39,-0.06) | 0.008 | 28.58 (5.77) | -0.06 (-0.20,-0.06) | 0.388 | 22.07 (8.22) | -0.26 (-0.50 -0.06) | 0.014 | 10.42 (5.65) | -0.05 (-0.18, 0.08) | 0.435 |
| **Number of children in house^5^** | 5.06 (2.25) | | 28.92 (6.42) | -0.30 (-0.48, 0.12) | 0.001 | 28.58 (5.77) | -0.06 (-0.22, 0.07) | 0.393 | 22.07 (8.22) | -0.38 (-0.61,-0.17) | 0.001 | 10.42 (5.65) | -0.07 (-0.21, 0.06) | 0.290 |
| **SCHOOL LEVEL** |  | |  |  |  |  |  |  |  |  |  |  |  |  |
| **Child teacher ratio**  15-34  35-44  45-54  55-64  ≥65 | 187 (15.2)  325 (26.4)  382 (31.1)  140 (11.4)  195 (15.9) | | 30.82 (5.27)  29.24 (5.98)  29.14 (7.12)  27.94 (5.65)  26.87 (6.47) | -1.58 (-3.76, 1.09)  -1.68 (-4.51, 1.37)  -2.89 (-5.03, 0.23)  -3.96 (-7.21, 0.06) | 0.132 | 29.04 (4.17)  29.12 (5.38)  29.50 (5.88)  28.09 (4.80)  25.82 (7.14) | 0.09 (-1.82, 1.94)  0.45 (-1.38, 2.13)  -0.95 (-2.52, 0.96)  -3.22 (-6.04,-0.25) | 0.125 | 23.76 (6.65)  22.42 (8.16)  22.94 (8.42)  20.84 (7.81)  19.16 (8.68) | -1.34 (-3.82, 1.75)  -0.83 (-3.60, 2.32)  -2.93 (-5.29,-0.22)  -4.61 (-7.80,-0.87) | 0.046 | 9.89 (5.58)  11.19 (5.78)  9.89 (5.92)  11.08 (4.51)  10.24 (5.47) | 1.30 (-0.26, 3.03)  -0.00 (-1.39, 1.30)  1.19 (-0.27, 2.43)  0.34 (-1.26, 1.74) | 0.186 |
| **School malaria control activities**  No  Yes | 915 (74.4)  314 (25.6) | | 28.77 (6.39)  29.38 (6.44) | 0.61 (-2.17, 2.99) | 0.642 | 28.26 (5.81)  29.51 (5.56) | 1.25 (-0.46, 2.93) | 0.168 | 22.06 (8.15)  22.17 (8.38) | 0.11 (-2.78, 3.01) | 0.940 | 10.34 (5.62)  10.68 (5.70) | 0.34 (-0.76, 1.80) | 0.600 |
| **School feeding programme**  No  Yes | 567 (46.1)  662 (53.9) | | 29.68 (6.71)  28.28 (6.05) | -1.40 (-3.41, 0.51) | 0.145 | 29.39 (5.44)  27.88 (5.95) | -1.51 (-3.06, 0.01) | 0.056 | 23.46 (8.05)  20.92 (8.15) | -2.55 (-4.78 -0.51) | 0.017 | 10.63 (5.72)  10.25 (5.57) | -0.38 (-1.39, 0.53) | 0.436 |
| **Administrative Division**  Diani  Lunga Lunga  Msambweni  Kubo | 327 (26.6)  494 (40.2)  161 (13.1)  247 (20.1) | | 30.93 (6.34)  28.17 (6.24)  28.11 (6.51)  28.31 (6.20) | -2.76 (-5.11,-0.13)  -2.82 (-5.22, 0.12)  -2.62 (-5.31,-0.01) | 0.083 | 30.54 (4.10)  28.08 (6.26)  28.86 (5.06)  26.80 (6.31) | -2.46 (-3.95,-0.87)  -1.68 (-3.55,-0.12)  -3.74 (-6.38,-1.90) | <0.001 | 25.15 (7.16)  20.71 (8.36)  21.67 (7.78)  21.07 (8.44) | -4.32 (-6.67,-2.15)  -3.48 (-6.10,-1.23)  -4.07 (-7.02,-1.22) | <0.001 | 11.12 (5.99)  10.01 (5.32)  11.28 (5.59)  9.80 (5.67) | -1.11 (-2.42, 0.14)  0.16 (-1.75, 1.90)  -1.32 (-2.60, 0.03) | 0.105 |

^1^ 1229 observations included for Silly Sentences test. 1219 observations included for Written Numeracy test. 1228 observations included for Spelling test. 1227 observations included for Code Transmission test. Percentage children per characteristic shown for 1229 children.

^2^All variables missing <3%

^3^Positive values indicate an increased score over reference group and negative values indicate a decreased score over reference group (95% CI is the bias corrected confidence interval)

^4^ P value is from multivariable Wald test derived from multivariable linear regression, bootstrapped and adjusted for school level clustering
